# Assessment of prevalence and factors affecting Mastalgia among early reproductive-aged women in Bangladesh: a cross-sectional survey

**DOI:** 10.1186/s12889-023-17173-7

**Published:** 2023-11-17

**Authors:** Abu Bakkar Siddique, Sudipto Deb Nath, Mahfuza Mubarak, Amena Akter, Sanjida Mehrin, Mst. Jemi Hkatun, Antara Parvine Liza, Md. Saiful Islam, M. Ziaul Amin, Most. Zannatul Ferdous

**Affiliations:** 1 Department of Public Health and Informatics, Jahangirnagar University, Savar, Dhaka, 1342 Bangladesh; 2 Centre for Advanced Research Excellence in Public Health, Savar, Dhaka, 1342 Bangladesh; 3 Army Medical College Jashore, Jashore, 7403 Bangladesh; 4 Department of Genetic Engineering & Biotechnology, Jashore University of Science & Technology, Jashore, 7408 Bangladesh; 5 Department of Agriculture, Bangabandhu Sheikh Mujibur Rahman Science & Technology University, Gopalganj, Dhaka, 8100 Bangladesh; 6 Department of Political Studies, Shahjalal University of Science & Technology, Sylhet, 3114 Bangladesh; 7 Shahjalal University of Science & Technology, Sylhet, 3114 Bangladesh

**Keywords:** Mastalgia, Early-reproductive age, Bangladesh, Prevalence, risk factors, Family history

## Abstract

**Background:**

Mastalgia, or breast pain, is a prevalent and distressing condition experienced by women, impacting their daily lives and causing complications. It is common among women of reproductive age, with associations found with premenstrual syndrome, fibrocystic breast disease, psychological distress, and, in rare cases, breast cancer. While Western societies have high frequencies of Mastalgia, it is less diagnosed in Asian cultures including Bangladesh. This study aimed to investigate the prevalence and factors associated with Mastalgia among early reproductive-aged women in Bangladesh.

**Methods:**

A cross-sectional survey was conducted, and data were collected from different regions of the country. A convenience sampling method was used to take 1,214 participants for the study. A pre-tested semi-structured questionnaire was used to collect data. Bivariate and multivariate analyses were conducted to ascertain factors that were significantly associated with Mastalgia. The data were analyzed using the SPSS software 26 version.

**Results:**

All the participants were female (mean age: 22.87 ± 2.64 years; age ranges: 18–35 years). The prevalence of Mastalgia was found to be 35.5%. The study was conducted using a self-reported semi-structured questionnaire. Participants with a higher education level and higher income were more likely to experience Mastalgia. A family history of breast cancer and severe abdominal pain during menstruation were also identified as associated factors for Mastalgia (p < 0.05).

**Conclusions:**

This study enhances our understanding of Mastalgia in Bangladesh, offering insights for healthcare and policy. Further research is required to uncover its root causes and develop effective interventions. The study highlights the prevalence of mastalgia and its related factors, emphasizing the necessity for increased awareness and support for affected women.

**Supplementary Information:**

The online version contains supplementary material available at 10.1186/s12889-023-17173-7.

## Introduction

Mastalgia, commonly known as breast pain, is a frequently encountered and distressing phenomenon among women. It exerts a significant impact on their daily lives, diminishing their quality of life and causing anxiety [1]. This condition is most commonly observed in women aged 15 to 40, a period considered to be within the reproductive age range [1, 2]. Up to two-thirds of women experience Mastalgia at some point during their reproductive years [3]. Mastalgia has been linked to premenstrual syndrome, fibrocystic breast disease, psychological distress, and, in rare instances, breast cancer. Additionally, extramammary conditions such as Tietze syndrome can also lead to Mastalgia [3, 4]. The pain is often described as a dull ache, although some women may encounter sensations of heaviness, tightness, discomfort, or a burning sensation in the breast tissue [4, 5].

Breast pain is typically categorized into two types: cyclic Mastalgia, and noncyclic Mastalgia [4]. It is the most commonly reported concern related to female breast health. Studies conducted in clinical settings have revealed that 67–75% of women under the age of 55 regularly experience breast discomfort before menstruation, and 11–30% report moderate to severe breast pain lasting for five days or more each month [3, 6]. Furthermore, in the United Kingdom, Mastalgia has been observed to have a prevalence as high as 34% among women in the early reproductive age group (18–36 years) due to increased sexual and reproductive activity during this period [7, 8].

In Western societies, Mastalgia, which refers to breast pain without any underlying pathology, is a common complaint that may affect up to 70% of women during their lifetime [9]. Interestingly, it is less prevalent in Asian cultures, affecting as few as 5% of women [10]. While it is not uncommon for women to experience mild breast pain for 2–3 days before menstruation, 8–30% of women report moderate to severe breast pain lasting for five or more days each month [9, 11, 12]. In Bangladesh, a previous study reported that general breast pain was the second most prevalent breast-related complication, with an overall prevalence of 24.22% [13].

The health-related consequences of Mastalgia are significant and difficult to fully articulate. According to certain research, Mastalgia has a detrimental impact on women’s daily activities and diminishes their overall health-related quality of life [10, 14]. In fact, 41% of women with Mastalgia reported a reduced quality of life and decreased sexual engagement, 35% reported poorer sleep quality, and 5% reported a diminished work-life balance [8]. Furthermore, the early reproductive age is a critical period during which women are more likely to engage in sexual and reproductive activities, and breast health is an essential component of their overall well-being [15]. Detecting Mastalgia and its determinants within this age range can contribute to early intervention and prevention strategies, potentially alleviating pain and discomfort [16]. Although numerous factors, including nutrition and psychological and hormonal factors, have been suggested, the exact cause has yet to be determined [17]. Therefore, it is imperative and pressing to investigate factors related to Mastalgia among women in their early reproductive years [18].

In Bangladesh, despite the high burden of breast cancer, little is known about the prevalence and factors affecting Mastalgia among early reproductive-aged women. Early reproductive age is a critical period for women’s reproductive health, and Mastalgia during this period may be indicative of underlying hormonal imbalances or other health conditions [18, 19]. However, the prevalence and risk factors associated with Mastalgia in this population are not well understood Breast-related complications like Mastalgia, Fibroadenoma are commonly underdiagnosed and undertreated in Bangladesh due to societal stigma and prejudice, as well as a scarcity of literature on the subject [20].

Moreover, there is no single study in Bangladesh exactly studying the factors and prevalence of Mastalgia simultaneously. The objective of this study was to examine the prevalence of Mastalgia and its associated factors, particularly those that are modifiable, among women in the early reproductive age group. So, this will be a great resource for our research community and policy makers to take adequate measures and interventions.

## Materials and methods

### Study area

The study was carried out in different parts of Bangladesh. Data was collected from Dhaka, Rajshahi, Chattogram, Sylhet and Barishal regions of Bangladesh which includes all the major regions of Bangladesh [21].

### Sample size

The sample size was calculated using the following equation:

$$n=\frac{{z}^{2}pq}{{d}^{2}}; n=\frac{{1.96}^{2}\times 0.24\times \left(1-0.24\right)}{{0.05}^{2}}=280.28\approx 281$$

Here,

*n* = number of samples

*z* = 1.96 (95% confidence level)

*p* = prevalence estimate (24.22% or 0.24) (based on a previous study)

*q* = (1-*p*)

*d* = Precession of the prevalence estimate (10% of 0.05)

We expected that the current study’s prevalence estimate (p) would be 24%. A sample size of 280.24 ≈ 281 people was estimated based on a 10% non-response rate. This estimate was exceeded by our sample size. However, 1214 participants were recruited to ensure the strength of the study.

### Study design, participants, and procedure

The present study employed a self-reported questionnaire-based cross-sectional survey design which was carried out from July to December 2022. The participants were enrolled using a convenience sampling technique. Each participant took approximately 10–15 min to complete the interview. Initially, 1310 participants attended the surveys. After removing incomplete responses, the final analysis included 1214 surveys. The data were gathered using a paper-based semi-structured questionnaire written in Bangla (the participant’s native language) from house to house.

A pilot test was carried out with 10 participants from the same population (target group) to determine the acceptability and transparency of the questionnaire. Following the pilot testing, a few minor adjustments were incorporated into the questionnaire. These data were not included in the final analysis. The first page of the questionnaire had an informed consent statement attached to it that explained the study’s objectives, procedures, and the participant’s right to decline participation. Before starting the survey, “participants were asked to obtain informed consent (i.e., *“Are you willing to participate in this study voluntarily and spontaneously?”*).The inclusion criteria of the participants included: i) Women at early reproductive age (18 to 35 years of age) [22], ii) experienced menstruation at their reproductive age [1], and iii) living in Bangladesh. The participants below 18 years and over 35 years were excluded at the time of the interview. As menstrual health is a very sensitive issue, the data was collected only by female research assistants and strict confidentiality was maintained.

### Measures

#### Socio-demographic measures and determinants of Mastalgia

Socio-demographic information was gathered by asking questions about age (later categorized as 18–24 years and 25–35 years) [22], marital status (married/ unmarried), education (below university/ university level), occupation (student/ unemployed/ employed/ others), family category (up to 4 members/ more than 4 members), monthly family income (less than 15,000 Bangladeshi Taka [BDT]/ 15,000 BDT to 30,000 BDT/ more than 30,000 BDT) [23] (109.73 BDT = 1 U$D [24]), Relationship status (in a relationship/ single), oral contraceptive [OCP] usage (yes/ no). (See questionnaire in Supplementary file).

#### Basic health-related measures and determinants of Mastalgia

Body Mass Index [BMI] (underweight/ normal weight/ overweight) (we measured the height and weight and calculated BMI), extra salt intake during meals (yes/ no), regular fast-food intake (yes/ no), smoking status(yes/ no), social media usage in a day (less than 2 h/ 2 to 5 h/ more than 5 h), soft drink intake (yes/ no), family history of breast cancer (yes/ no), bra usage (yes/ no), and large bra usage (yes/ no) [1, 25].

#### Menstruation-related measures and determinants of Mastalgia

The menstruation starting age (8 to 11 years/ 12 to 14 years/ more than 14 years), type of menstruation (regular/irregular), the average duration of menstruation (less than 3 days/3 to 6 days/ 7 or more days), amount of blood during menstruation (comparatively low/ normal/high), abdominal pain during menstruation (severe pain/ moderate pain/ no pain), family history of Mastalgia (yes/ no) [1].

#### Characteristics of Mastalgia and its effect on daily life

Type of breast pain (periodic breast pain/ non-periodic breast pain), pain in both breast (yes/no), pain in both breast (both breast/ one breast), location of breast pain (entire breast/ In a certain area), fever during breast pain (yes/ no), breast pain goes after menstruation starts(yes/no), effects of this pain on daily activities (yes/ no), effect of this pain on daily activities (yes/no), characteristics of the pain (tingling/ throbbing/ stinging/ burning/ cramping/ crushing/ tugging), consulted a doctor for this pain (yes/ no), regular follow-ups (yes/ no), type of diagnostic method used (ultrasonogram/ clinical test or size test/ mammography/ others/ didn’t conduct any diagnostics) multiple responses were taken to this question, The intensity of pain was determined with the aid of a scale graded from 0 to 10, to be very severe at 10 and none at 0 [1, 25].

#### Dependent variable

The presence of Mastalgia was identified by self-perception of the respondents. The interviewer asked participants, “*Do you have swelling and tenderness during or immediately preceding menstruation, as well as breast tissue pain / swelling and tenderness outside of the menstrual period (yes/ no) ?* ” [1].

### Statistical analysis

All statistical analyses were performed using Microsoft Excel 2019 and SPSS version 26.0 (Chicago, IL, USA). Descriptive statistics, such as frequencies, percentages, averages, and standard deviations (SDs), were computed. Bivariate logistic regression analysis and multivariable logistic regression analysis were used to determine the connection between the dependent and independent variables. Factors found to be significant in the bivariate logistic regression analysis were eventually incorporated in the multivariable logistic regression analysis. For all analyses, a p-value of less than 0.05 was considered statistically significant.

### Ethical consideration

The survey was carried out in accordance with the Helsinki Declaration of 1975. The Ethics Review Committee of the Faculty of Biological Science and Technology, Jessore University of Science and Technology, Jessore-7408, Bangladesh [Ref: ERC/FBST/JUST/2022-l-0 L, Date: 13/02/2022] examined and approved the study protocol. All respondents were informed of the study’s goal, procedure, and ability to withdraw their data. Prior to completing the study, each participant provided written informed consent. Participants were advised that all of their information would be kept anonymous and confidential, and they were given information regarding the study’s nature and goal.

## Result

### General characteristics of participants

A total of 1214 participants (female: 100%) were included in the final analysis. Most of them (87.4%) were aged between 18 and 24 years and 83.9% of them are unmarried. The majority were students (87.1%), had university-level educated (91.4%), were from nuclear families (79.2), came from urban areas (82.2%), and had a family monthly income of more than 30,000 Bangladeshi Taka (BDT; 103.51 BDT = 1US$) (45.9%). Most of the respondents 71.9% are single and didn’t use OCP (oral contraceptive pill).

Almost every (96.6%) respondent had normal body mass index (BMI), while 50.9% took fast food regularly, 42.1% took extra salt in meals, 55.2% drank soft drinks, and 3.2% of respondents smoked. The majority of respondents had no family history of breast cancer (84.3%), used a bra sometimes in a day (64.3%), opted for a perfectly fitting bra (88.0%), and spent 2 to 5 h per day on social media (58.2%). Furthermore, 76.7% of participants reported having regular menstruation, while 74.6% experienced a normal blood flow during menstruation. Most respondents had a menstrual duration lasting between 3 and 6 days (75.1%), with menstruation typically commencing between the ages of 12 to 14 years (75.7%), and moderate abdominal pain occurring during menstruation (58.3%). Notably, 72.5% of the respondents did not have a family history of mastalgia. The overall prevalence of Mastalgia was 35.5% (Table 1).

**Table 1:** General characteristics of the participants (N = 1,214)

Variables	n (%)
**Socioeconomic information**	
**Age**	
18 to 24 years	1061 (87.4)
25 to 35 years	153 (12.6)
**Marital status**	
Unmarried	1018 (83.9)
Married	196 (16.1)
**Education level**	
Below university	105 (8.6)
University level	1109 (91.04)
**Occupation**	
Student	1058 (87.1)
Unemployed	34 (2.8)
Employed	63 (5.2)
Others	59 (4.9)
**Place of residence**	
Rural	216 (17.8)
Urban	998 (82.2)
**Family Type**	
Nuclear (up to 4 members)	961 (79.2)
Large (more than 4 members)	253 (20.8)
**Monthly family income**	
< 15,000 BDT	242 (19.9)
15,000–30,000 BDT	415 (34.2)
> 30,000 BDT	557 (45.9)
**Relationship status**	
In a relationship	341 (28.1)
Single	873 (71.9)
**OCP Usage**	
Yes	73 (6.0)
No	1141 (94.0)
**Basic health-related information**	
**BMI**	
Underweight	192 (15.8)
Normal	840 (96.2)
Overweight	182 (15.2)
**Extra salt intake during meals**	
Yes	511 (42.1)
No	703 (57.9)
**Regular fast-food intake**	
Yes	618 (50.9)
No	596 (49.1)
**Smoking**	
Yes	39 (3.2)
No	1175 (96.8)
**Social media usage in a day**	
Less than 2 h	271 (22.3)
2 to 5 h	706 (58.2)
More than 5 h	237 (19.5)
**Soft drink intake**	
Yes	670 (55.2)
No	544 (44.8)
**Family history of breast cancer**	
Yes	191 (15.7)
No	1023 (84.3)
**Bra usage**	
No	132 (10.9)
Sometimes	780 (64.3)
Everyday	302 (24.9)
**Large Bra usage**	
Yes	146 (12.0)
No	1068 (88.0)
**Menstruation related factors**	
**Menstruation starting age**	
8 to 11 years	199 (16.4)
12 to 14 years	910 (75.0)
More than 14 years	105 (8.6)
**Type of menstruation**	
Regular	931 (76.7)
Irregular	283 (23.3)
**Average duration of menstruation**	
Less than 3 days	138 (11.4)
3 to 6 days	912 (75.1)
7 or more days	164 (13.5)
**Amount of blood during menstruation**	
Comparatively Low	178 (14.7)
Normal	906 (74.6)
High	130 (10.7)
**Abdominal pain during menstruation**	
Severe pain	352 (29.0)
Moderate pain	708 (58.3)
No pain	154 (12.7)
**Family History of Mastalgia**	
Yes	334 (27.5)
No	880 (72.5)

### Descriptive characteristics of Mastalgia

35.5% of the respondents reported that they had Mastalgia. Among those who experienced breast pain, the majority (74.9%) reported having non-periodic breast pain. The majority of respondents who consulted a doctor for breast pain constituted 19.3% of the total. In the study, women reporting pain severity assessed it to be 4.84 ± 2.29 out of 10. The majority of participants experienced pain in both breasts (69.4%). When considering the location of pain, the majority experienced pain throughout the entire breast (51.7%), and 8.8% respondents experienced fever during the pain. In terms of the impact of breast pain, 29.7% reported to have effect on daily activities, while 22.5% reported effect on sleep. Among the characteristics of the pain, the majority of respondents (43.2%) described it as tugging. Furthermore, regular follow-ups were reported by a minority of respondents (8.1%). Only 26.9% respondents conducted any diagnostic tests for determining the cause of the pain (Table 2).

**Table 2:** Descriptive Characteristics of Mastalgia

Variables	n (%)
**Experience of breast pain (Mastalgia)**	
No	783 (64.5)
Yes	431 (35.5)
**Type of breast pain**	
Periodic breast pain	108 (25.1)
Non periodic breast pain.	323 (74.9)
**The severity and nature of breast pain**	
(n = 431) (x ± SD)	4.84 (2.295)
**Pain in both breast**	
Both breast	299 (69.4)
One breast	132 (30.6)
**Location of pain**	
Entire breast	223 (51.7)
In a certain area	208 (48.3)
**Fever during pain**	
Yes	38 (8.8)
No	393 (91.2)
**Breast pain goes after the menstruation starts**	
Yes	331 (76.8)
No	100 (23.2)
**Effect of this pain on daily activities**	
Yes	128 (29.7)
No	303 (70.3)
**Effect of this pain on sleep**	
Yes	97 (22.5)
No	334 (77.5)
**Characteristics of the pain**	
Tingling	99 (23.0)
Throbbing	16 (3.7)
Stinging	84 (19.6)
Burning	18 (4.2)
Cramping	8 (1.9)
Crushing	20 (4.6)
Tugging	186 (43.2)
**Consulted a doctor for breast pain**	
Yes	83 (19.3)
No	348 (80.7)
**Regular follow-ups**	
Yes	35 (8.1)
No	396 (91.9)
**Type of diagnostic tests used**	
Ultrasonogram	56 (13.0)
Clinical test/Size test	42 (9.7)
Mammography	12 (2.8)
Others	6 (1.4)
Didn’t conduct any diagnostics	315 (73.1)

### Regression analysis

Table 3 shows the result of binary logistic regression analysis by self-reported Mastalgia occurrence. All the variables were included in the adjusted models. As per the adjusted binary logistic analysis, participants from the below university group were less likely to have Mastalgia compared to the University level group (AOR = 0.57, 95% CI = 0.34–0.98, p = 0.044). Higher odds of Mastalgia were found among participants who had monthly family income of more than 30,000 BDT (AOR = 1.50, 95% CI = 1.02–2.22, p = 0.037) compared to the lower income group (less than 15,000 BDT).

**Table 3:** Binary and multiple regression analysis of factors associated with Mastalgia

Variables	Yes (%)	NO (%)	Unadjusted model		Adjusted model^a^	
			COR (95% CI)	*p*-value	AOR (95% CI)	*p*-value
**Socioeconomic information**						
**Age**						
18 to 24 years	370 (30.5)	691 (56.9)	Reference		Reference	
25 to 35 years	61 (5.0)	92 (7.6)	1.23 (1.036–1.931)	0. 0.228	0.93 (0.55–1.57)	0.800
**Marital status**						
Unmarried	348 (28.7)	670 (55.2)	Reference		Reference	
Married	83 (6.8)	113 (9.3)	1.41 (0.51–0.96)	**0.029**	1.09 (0.69–1.72)	0.696
**Education level**						
Below university	29 (2.4)	76 (6.3)	0.67 (0.43–1.04)	0.079	0.57 (0.34–0.98)	**0.044**
University level	402 (33.1)	707 (58.2)	Reference		Reference	
**Occupation**						
Student	369 (30.4)	689 56.8)	Reference		Reference	
Unemployed	8 (0.7)	26 (2.1)	0.57 (0.25–1.28)	0.176	0.71 (0.27–1.82)	0.480
Employed	32 (2.6)	31 (2.6)	1.92 (1.15–3.20)	**0.012**	1.34 (0.67–2.71)	0.402
Others	22 (1.8)	37 (3.0)	1.11 (0.64-1.91)	0.706	1.34 (0.59–3.03)	0.476
**Place of residence**						
Rural	66 (5.4)	150 (12.4)	0.76 (0.55–1.04)	0.094	1.39 (0.98–1.97)	0.063
Urban	365 (30.1)	633 (52.1)	Reference		Reference	
**Family Type**						
Nuclear	349 (28.7)	612 (50.4)	1.18 (0.88–1.59)	0.248	0.91 (0.63–1.31)	0.624
Large	82 (6.8)	171 (14.1)	Reference		Reference	
**Monthly family income**						
< 15,000 BDT	66 (5.4)	176 (14.5)	Reference		Reference	
15,000–30,000 BDT	146 (12.0)	269 (22.2)	1.44 (1.02–2.04)	**0.037**	1.32 (0.89–1.95)	0.167
> 30,000 BDT	219 (18.0)	338 (27.8)	1.72 (1.24–2.40)	**0.001**	1.50 (1.02–2.22)	**0.037**
**Relationship status**						
Yes	147 (12.1)	194 (16.0)	1.57 (1.21–2.03)	**0.001**	1.29 (0.95–1.76)	0.095
No	284 (23.4)	589 (48.5)	Reference		Reference	
**OCP Usage**						
Yes	34 (2.8)	39 (3.2)	1.63 (1.015–2.62)	**0.043**	0.90 (0.46–1.75)	0.759
No	397 (32.7)	744 (61.3)	Reference		Reference	
**Basic health related information**						
**BMI**						
Underweight	58 (4.8)	134 (11.0)	Reference		Reference	
Normal	312 (25.7)	528 (43.5)	1.36 (0.97–1.91)	0.071	1.16 (0.79–1.70)	0.444
Overweight	61 (5.0)	121 (10.0)	1.16 (0.75–1.80)	0.493	0.81 (0.48–1.34)	0.420
**Extra salt intake**						
Yes	201 (16.6)	310 (25.5)	1.33 (1.05–1.69)	**0.017**	1.26 (0.96–1.66)	0.091
No	230 (18.9)	473 (39.0)	Reference		Reference	
**Regular fast-food intake**						
Yes	240 (19.8)	378 (31.1)	1.34 (1.06–1.70)	**0.014**	1.02 (0.75–1.38)	0.889
No	191(15.7)	405 (33.4)	Reference			
**Smoking**						
Yes	20 (1.6)	19 (1.6)	1.95 (1.03–3.70)	**0.040**	1.13 (0.53–2.42)	0.740
No	411 (33.9)	764 (62.9)	Reference		Reference	
**Social media usage in a day**						
Less than 2 h	77 (6.3)	194 (16.0)	Reference		Reference	
2 to 5 h	256 (21.1)	450 (37.1)	1.43 (1.05–1.94)	**0.021**	1.15 (0.81–1.63)	0.434
More than 5 h	98 (8.1)	139 (11.4)	1.776 (1.228–2.57)	**0.002**	1.35 (0.88–2.07)	0.162
**Soft drink intake**						
Yes	268 (22.1)	402 (33.1)	1.55 (1.22–1.98)	**< 0.001**	1.29 (0.95–1.76)	0.100
No	163 (13.4)	381 (31.4)	Reference		Reference	
**Family history of breast cancer**						
Yes	97 (8.0)	94 (7.7)	2.12 (1.55–2.90)	**< 0.001**	1.80 (1.26–2.58)	**0.001**
No	334 (27.5)	689 (56.8)	Reference		Reference	
**Bra usage**						
No	37 (3.0)	95 (7.8)	0.94 (0.60–1.49)	0.814	0.97 (0.57–1.63)	0.917
Sometimes	306 (25.2)	474 (39.0)	1.57 (1.17–2.09)	**0.002**	1.42 (1.03–1.96)	0.029
Everyday	88 (7.2)	214 (17.6)	Reference			
**Large Bra usage**						
Yes	68 (5.6)	78 (6.4)	1.69 (1.19–2.40)	**0.003**	1.24 (0.83–1.87)	0.284
No	363 (29.9)	705 (58.1)	Reference		Reference	
**Menstruation related factors**						
**Menstruation starting age**						
8 to 11 years	80 (6.6)	119 (9.8)	1.94 (1.15–3.27)	**0.013**	1.48 (0.83–2.66)	0.180
12 to 14 years	324 (26.7)	586 (48.3)	1.59 (1.01–2.52)	**0.045**	1.42 (0.86–2.35)	0.170
More than 14 years	27 (2.2)	78 (6.4)	Reference		Reference	
**Type of menstruation**						
Regular	327 (26.9)	604 (49.8)	Reference		Reference	
Irregular	104 (8.6)	179 (14.7)	1.07 (0.81–1.41)	0.617	0.94 (0.67–1.32)	0.734
**Duration of menstruation**						
Less than 3 days	61 (5.0)	77 (6.3)	1.48 (0.93–2.36)	0.094	0.83 (0.44–1.55)	0.565
3 to 6 days	313 (25.8)	599 (49.3)	0.98 (0.69–1.39)	0.981	0.85 (0.55–1.29)	0.448
7 or more days	57 (4.7)	107 (8.8)	Reference		Reference	
**Amount of blood during menstruation**						
Comparatively Low	80 (6.6)	98 (8.1)	Reference		Reference	
Normal	297 (24.5)	609 (50.2)	0.59 (0.43 − 0.08)	**0.002**	0.66 (0.42–1.03)	0.068
High	54 (4.4)	76 (6.3)	0.87 (0.55–1.37)	0.552	0.80 (0.43–1.47)	0.483
**Abdominal pain during menstruation**						
Severe pain	157 (12.9)	195 (16.1)	2.639 (1.72–4.050)	**< 0.001**	2.40 (1.49–3.87)	**< 0.001**
Moderate pain	238 (19.6)	470 (38.7)	1.66 (1.10–2.48)	**0.014**	1.48 (0.95–2.31)	0.078
No pain	36 (3.0)	118 (9.7)	Reference		Reference	
**Family History of Mastalgia**						
Yes	209 (17.2)	125 (10.3)	4.95 (3.78–6.48)	**< 0.001**	4.37 (3.27–5.83)	**< 0.001**
No	222 (18.3)	658 (54.2)	Reference		Reference	

Notes: COR = Unadjusted/ Crude odds ratio; CI = Confidence interval; AOR = Adjusted odds ratio

Participants having a family history of breast cancer were approximately two times more likely to have Mastalgia compared to those having no family history (AOR = 1.80, 95% CI = 1.26–2.58, p = 0.001). Those who had severe abdominal pain during the menstruation were two times more likely to have Mastalgia compared to those who didn’t have any pain during the menstruation (AOR = 2.40, 95% CI = 1.49–3.87, p = < 0.001). In addition, participants who had a family history of Mastalgia were four times more likely to have Mastalgia compared to those having no family history (AOR = 4.37, 95% CI = 3.27–5.83, p = < 0.001).

**Fig. 1:**
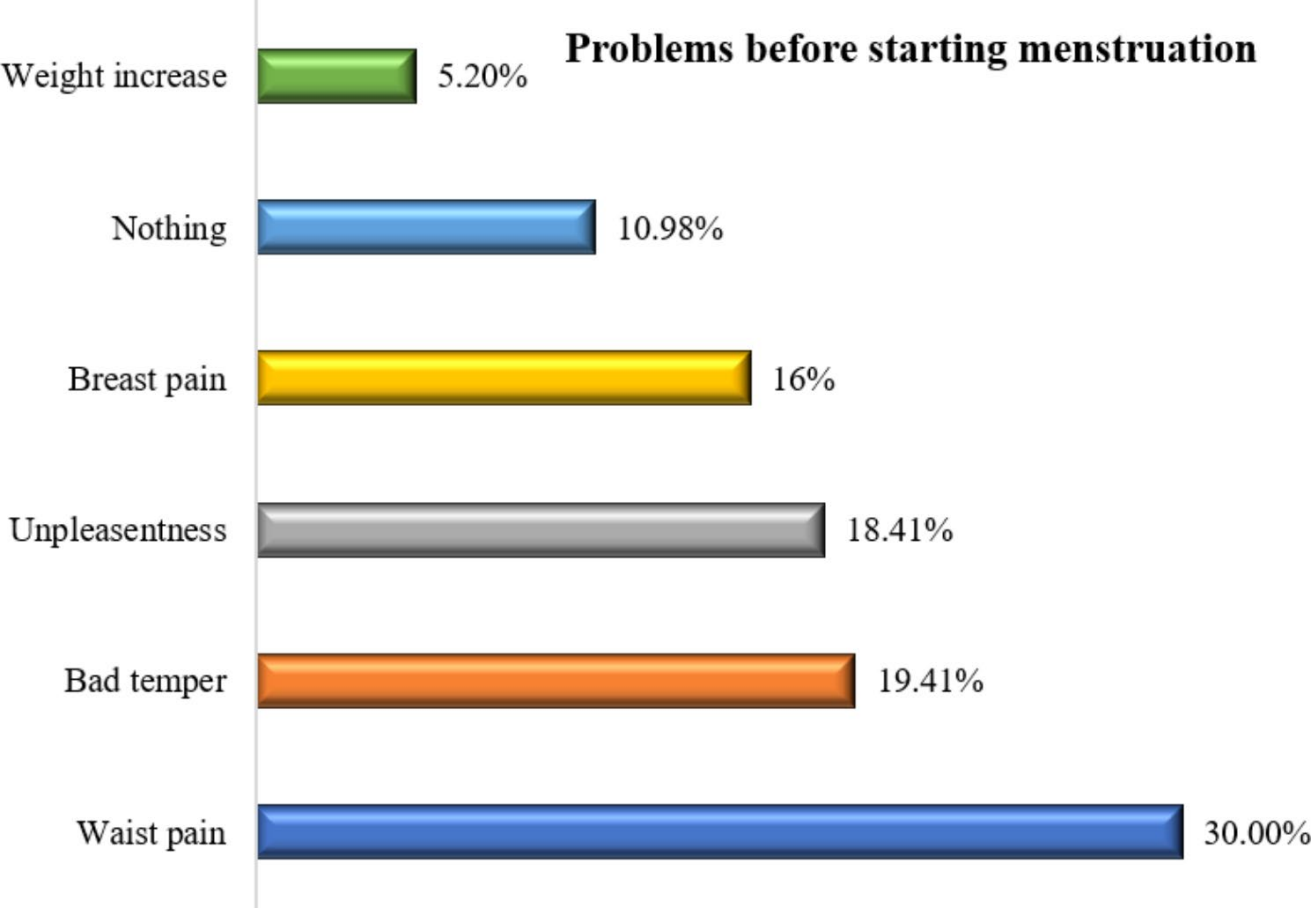
Problems before starting menstruation

Figure 1 depicts the problems before starting menstruation. 30% and 19.41% of the respondents reported that they experienced waist pain and bad temper respectively.

## Discussion

Breast pain known as Mastalgia arises from the breast tissue and is one of the most common breast symptoms among reproductive-aged women [2]. Its impact on quality of life is frequently underestimated and may be severe enough to interfere with routine everyday tasks all over the country, including Bangladesh [10, 26]. Mastalgia, considered one of our country’s major public health issues, is the leading common breast-related problem among women in both developed and developing countries, including Bangladesh. Adjusted binary logistic analysis revealed significant associations: lower university group participants had lower odds of Mastalgia compared to those at the university level; individuals with a monthly family income exceeding 30,000 BDT had higher odds of Mastalgia compared to the lower-income group; participants with a family history of breast cancer were approximately twice as likely to experience Mastalgia as those without such a history; individuals with severe menstrual abdominal pain had a higher likelihood of Mastalgia compared to those without pain; and those with a family history of Mastalgia were more likely to have Mastalgia compared to those without such a history.

Our study revealed that about one-third (35.5%) of the participants had experienced Mastalgia. There is no single study investigating the associated factors of Mastalgia in Bangladesh. Other studies in different parts of the world exploring Mastalgia show varying data. For example, in India, 47.33% of women experience Mastalgia [27], compared to 33% in Iran [25]. About 68% of women in the USA, 51.5% of women in Canada, and 32% in the UK have been reported to experience Mastalgia [27]. The prevalence of Mastalgia in Sub-Saharan Africa and China are 16% and 45.3% respectively [28, 29]. Factors associated with Mastalgia include higher age, university graduate, BMI of > 30, excessive use of salt, use of a small bra, and use of a large bra were in line with Turkish study [30].

In our study, 2.4% of the below university level & 33.1% of the university level suffer from Mastalgia and university graduates are more prone to Mastalgia. This is in line with Egyptian and Turkish studies where graduates from university suffered more from Mastalgia [28, 30]. It’s not clear if is there any relationship between Mastalgia and education level. Further study is needed to know what is the cause of Mastalgia at the higher education level.

The study results are consistent with other studies that have reported an association between socioeconomic status and Mastalgia. For instance, a study conducted in the United States reported a higher prevalence of Mastalgia among women with higher income and education levels [31]. On the other hand, a study conducted in Iran reported no association between Mastalgia among women and socioeconomic status [32].

The findings of the study also suggest that genetic factors may play a role in Mastalgia occurrence. Women with a family history of breast cancer or Mastalgia were more likely to have Mastalgia. These findings are consistent with other studies that have reported an association between a family history of breast cancer and Mastalgia [33]. Another study in Iraq also found that there is a strong relationship positive family history of breast cancer and Mastalgia [34]. There is compelling evidence that breast cancer can lead to breast pain, establishing a connection between Mastalgia and breast edema and stroma [33].

Furthermore, the study results highlight the importance of severe abdominal pain during the menstruation as a risk factor for Mastalgia. Previous studies have also reported an association between menstrual pain and Mastalgia. A study conducted in Saudi Arabia reported a higher prevalence of Mastalgia among women who experienced severe menstrual pain [35]. Additionally, Mastalgia is highly related to menstruation [1].

Furthermore, we found an association between a family history of Mastalgia and Mastalgia cases. This is a similar finding according to a prospective study of turkey [33], indicating a clear relationship between genetic factors and Mastalgia [36].

In our study, we didn’t find any relationship between Mastalgia who had a history of smoking. But there are some studies where they found a close relationship between Mastalgia who had a smoking history [36, 37]. Further study is needed to clarify of this association.

Some studies found positive association between Mastalgia and BMI [30, 38]. But in our, we didn’t find any relationship between BMI and Mastalgia. So, the environmental cause may also be related to Mastalgia. Further study is needed to evaluate Mastalgia that is there any association that leads to Mastalgia.

### Limitations

It is worth noting that the study has a few limitations. One of the main limitations is that the study relies on self-reported data, which may introduce bias or error. Participants may have different interpretations of what constitutes Mastalgia or may be more or less likely to report symptoms based on factors such as cultural beliefs or social desirability bias. Another limitation is that the study is cross-sectional, which means that causality cannot be inferred. It is not possible to know whether the risk factors identified in this study directly cause Mastalgia or whether they are simply associated with it. Furthermore, the study was conducted in Bangladesh, and the results may not be generalizable to other populations.

## Conclusion

Despite these limitations, the study’s findings highlight several potential associated factors for Mastalgia, including family history of breast cancer or Mastalgia, severe abdominal pain during menstruations, higher income, and higher educational status. Clinicians may want to consider these factors when assessing patients who report symptoms of Mastalgia. Furthermore, the findings suggest that public health interventions may be needed to reduce the burden of Mastalgia among certain populations, such as those with a family history of breast cancer or those with severe menstrual pain.

### Electronic supplementary material

Below is the link to the electronic supplementary material.

Supplementary Material 1

## Data Availability

The authors state that they have no financial and non-financial potential conflicts interests of interest in publishing the results of their research.

## List of abbreviations

AOR: Adjusted Odds Ratio
BDT: Bangladeshi Taka
BMI: Body Mass Index
CI: Confidence Interval
MH: Menstrual Health
OCP: Oral Contraceptive Pill
SD: Standard Deviation
SPSS: Statistical Package for the Social Sciences

## References

[1] Koçoğlu D, Kurşun S, Akın B, Altuntug K. “Mastalgia and associated factors: a cross-sectional study.,” *Agri Agri Dernegi’nin Yayin organidir = J. Turkish Soc. Algol*, vol. 29, no. 3, pp. 100–108, Jul. 2017, 10.5505/agri.2017.91069 10.5505/agri.2017.91069 29039149

[2] “Mastalgia NCBI. ” 2022. https://www.ncbi.nlm.nih.gov/books/NBK562195/

[3] Tahir MT, Shamsudeen S. “Mastalgia.,” Treasure Island (FL), 2023. [Online]. Available: https://europepmc.org/article/nbk/nbk562195

[4] Olawaiye A, Withiam-Leitch M, Danakas G, Kahn K. “Mastalgia: a review of management.,” *J. Reprod. Med*, vol. 50, no. 12, pp. 933–939, Dec. 2005. 16444894

[5] Berens PD. “Breast Pain: Engorgement, Nipple Pain, and Mastitis.,” *Clin. Obstet. Gynecol*, vol. 58, no. 4, pp. 902–914, Dec. 2015, 10.1097/GRF.0000000000000153 10.1097/GRF.0000000000000153 26512442

[6] Ader DN, South-Paul J, Adera T, Deuster PA. “Cyclical mastalgia: prevalence and associated health and behavioral factors.,” *J. Psychosom. Obstet. Gynaecol*, vol. 22, no. 2, pp. 71–76, Jun. 2001, 10.3109/01674820109049956 10.3109/01674820109049956 11446156

[7] Davies EL, Gateley CA, Miers M, Mansel RE. “The long-term course of mastalgia.,” *J. R. Soc. Med*, vol. 91, no. 9, pp. 462–464, Sep. 1998, 10.1177/014107689809100903 10.1177/014107689809100903 PMC1296872 9849515

[8] Scurr J, Hedger W, Morris P, Brown N (2014). The prevalence, severity, and impact of breast pain in the general population. ” Breast J.

[9] Ernster VL et al. “Effects of caffeine-free diet on benign breast disease: a randomized trial.,” *Surgery*, vol. 91, no. 3, pp. 263–267, Mar. 1982. 7058508

[10] Salzman B, Collins E, Hersh L. “Common Breast Problems.,” *Am. Fam. Physician*, vol. 99, no. 8, pp. 505–514, Apr. 2019. 30990294

[11] Ader DN, Browne MW (1997). Prevalence and impact of cyclic mastalgia in a United States clinic-based sample. Am J Obstet Gynecol.

[12] Salzman B, Fleegle S, Tully AS. “Common breast problems.,” *Am. Fam. Physician*, vol. 86, no. 4, pp. 343–349, Aug. 2012. 22963023

[13] Ahmed S, Awal A (2018). Spectrum of benign breast Diseases in Females- a 10 years study. Med Today.

[14] Santen RJ, Mansel R. “Benign breast disorders.,” *N. Engl. J. Med*, vol. 353, no. 3, pp. 275–285, Jul. 2005, 10.1056/NEJMra035692 10.1056/NEJMra035692 16034013

[15] Ibitoye M, Choi C, Tai H, Lee G, Sommer M (2017). Early menarche: a systematic review of its effect on sexual and reproductive health in low- and middle-income countries. ” PLoS One.

[16] Ginsburg O et al. “Breast cancer early detection: A phased approach to implementation.,” *Cancer*, vol. 126 Suppl 10, no. Suppl 10, pp. 2379–2393, May 2020, 10.1002/cncr.32887 10.1002/cncr.32887 PMC7237065 32348566

[17] Kataria K, Dhar A, Srivastava A, Kumar S, Goyal A. “A systematic review of current understanding and management of mastalgia.,” *Indian J. Surg*, vol. 76, no. 3, pp. 217–222, Jun. 2014, 10.1007/s12262-013-0813-8 10.1007/s12262-013-0813-8 PMC4141056 25177120

[18] Akter MF, Ullah MO. “Awareness levels of breast cancer among female university and medical college students in Sylhet city of Bangladesh.,” *Cancer reports (Hoboken, N.J.)*, vol. 5, no. 11, p. e1608, Nov. 2022, 10.1002/cnr2.1608 10.1002/cnr2.1608 PMC9675358 35122415

[19] Musey VC, Collins DC, Musey PI, Martino-Saltzman D, Preedy JRK (1987). Age-related changes in the female hormonal environment during reproductive life. Am J Obstet Gynecol.

[20] Steiness HS, Villegas-Gold M, Parveen H, Ferdousy T, Ginsburg O. Barriers to care for women with Breast cancer symptoms in rural Bangladesh. ” Health Care Women Int. May 2018;39(5):536–54. 10.1080/07399332.2018.1446958 10.1080/07399332.2018.1446958 29505392

[21] Wikipedia. Regions of Bangladesh, 2020. https://en.wikipedia.org/wiki/Administrative_geography_of_Bangladesh

[22] Svetlana E, Natalia A, Anzhelika B (2019). Indicators of an ovarian reserve in women of early reproductive age with PCOS depending on the phenotype. Horm Mol Biol Clin Investig.

[23] Islam MS (2021). Knowledge, attitudes and perceptions towards COVID-19 vaccinations: a cross-sectional community survey in Bangladesh. BMC Public Health.

[24] Google “BDT, to USD. ” 2023. https://www.google.com/search?q=1%24+to+taka&oq=1%24&aqs=chrome.1.69i57j0i131i433i512j0i512l8.3528j0j4&sourceid=chrome&ie=UTF-8.

[25] Vaziri F, Samsami A, Rahimi Z, Rastgardoost N, Nick N (2016). Prevalence, severity and factors related to Mastalgia among women referring to Health centers affiliated with Shiraz University of Medical Sciences. J Heal Sci Survellience Syst.

[26] Kanat BH, et al. Effects of Mastalgia in Young Women on Quality of Life, Depression, and anxiety levels. Indian J Surg. Apr. 2016;78(2):96–9. 10.1007/s12262-015-1325-5 10.1007/s12262-015-1325-5 PMC4875894 27303116

[27] Raghunath S, Raghuram N, Ravi S, Ram N, Ram A (2015). Prevalence of mastalgia in young Indian females. J Heal Res Rev.

[28] Makumbi T, Galukande M, Gakwaya A. “Mastalgia: prevalence at a sub-saharan african tertiary hospital.,” *Pain Res. Treat*, vol. 2014, p. 972726, 2014, 10.1155/2014/972726 10.1155/2014/972726 PMC4199068 25349735

[29] Thomas DB et al. “Randomized trial of breast self-examination in Shanghai: final results.,” *J. Natl. Cancer Inst*, vol. 94, no. 19, pp. 1445–1457, Oct. 2002, 10.1093/jnci/94.19.1445 10.1093/jnci/94.19.1445 12359854

[30] Koçoglu D, Kursun S, Akin B, Altuntug K (2017). Mastalgia and associated factors: a cross-sectional study. Agri.

[31] Barton MB, Elmore JG, Fletcher SW. Breast symptoms among women enrolled in a health maintenance organization: frequency, evaluation, and outcome. ” Ann Intern Med. Apr. 1999;130(8):651–7. 10.7326/0003-4819-130-8-199904200-00005 10.7326/0003-4819-130-8-199904200-00005 10215561

[32] Shobeiri F, Oshvandi K, Nazari M (2016). Cyclical mastalgia: prevalence and associated determinants in Hamadan City, Iran. Asian Pac J Trop Biomed.

[33] Yıldırım AC, Yıldız P, Yıldız M, Kahramanca Ş, Kargıcı H. Mastalgia-Cancer relationship: a prospective study. ” J Breast Heal. Apr. 2015;11(2):88–91. 10.5152/tjbh.2015.2492 10.5152/tjbh.2015.2492 PMC5351493 28331698

[34] Mohammed AA (2020). Evaluation of mastalgia in patients presented to the breast clinic in Duhok city, Iraq: Cross sectional study. Ann Med Surg.

[35] AlJbeery AS, AlShaifani AA, AlMutlaq SM, Salati SA (2017). Mastalgia-A study from the Middle East. Online J Heal Allied Sci.

[36] Eren T et al. “Factors Effecting Mastalgia.,” *Breast Care (Basel)*, vol. 11, no. 3, pp. 188–193, Jun. 2016, 10.1159/000444359 10.1159/000444359 PMC4960349 27493619

[37] Hafiz SP, Barnes NLP, Kirwan CC. “Clinical management of idiopathic mastalgia: a systematic review.,” *J. Prim. Health Care*, vol. 10, no. 4, pp. 312–323, Dec. 2018, 10.1071/HC18026 10.1071/HC18026 31039960

[38] Sabry R, Kolib TM, Ahmed M, Elnahas HG (2021). Body mass index and other factors related to mastalgia: a cross sectional study. Open Access Maced J Med Sci.

