# Supplementary material for: Assessment of prevalence and factors affecting Mastalgia among early reproductive-aged women in Bangladesh: a cross-sectional survey
Source: BMC Public Health. 2023 Nov 17;23:2269. doi: 10.1186/s12889-023-17173-7 (PMC10656957; doi:10.1186/s12889-023-17173-7)
Supplement: Supplementary file 1 — Supplementary Material 1 [file 12889_2023_17173_MOESM1_ESM.docx]

**Assessment of prevalence and factors affecting Mastalgia among early reproductive-aged women in Bangladesh: A cross-sectional survey.**

There is not much research in Bangladesh on the physical health of girls. Moreover, there is no study investigating mastalgia in Bangladesh. So, we are going to find out the prevalence and factors affecting Mastalgia among early reproductive-aged women in Bangladesh.

It will take only 10-12 minutes to participate in this study, and this precious 10-12 minutes of your time is very important for our research. No one's name, phone number, or address will be taken here, so there will be no problem with privacy. Therefore, if you are willing to help with our research, please answer the following questions correctly. However, do not give any wrong or false information or tick without ✔ reading the question. Thank you.

Are you willing to participate in our study? A) Yes B) No

Research Assistant’s name………………………

Date……………….

Serial No……….

**Section A: Socio-demographic Information**

| A1. How old are you? | **.......................................** |
| --- | --- |
| A2. What is your marital status? | a) Unmarried  b) Married  c) Divorce/Divorce |
| A3. What's your job? | a) Student  b) Unemployed  c) Employed  d) Others |
| A4. What is your educational qualification? | 1. Below university 2. University level |
| A5. Where is your permanent address? | 1. Village 2. City |
| A6. What is the type of family? | 1. Single 2. joint |
| A7. Total monthly income of the family (Taka)? | a) <15,000 BDT  b) 15,000-30,000 BDT  c) > 30,000 BDT |
| A8. Are you in a relationship? | a) Yes  b) No |
| A9. Do you take birth control pills/ Oral contraceptive pill? | a) Yes  b) No |

**Section B: Basic Health-related Information**

| B1. Height? | ......... ....feet,... .. ....inches. |
| --- | --- |
| B2. Weight | ..................... (kg) |
| B3. Do you use raw salt with food? | a) Yes  b) No |
| B4. Do you often eat fast food? | a) Yes  b) No |
| B5. Do you smoke? | a) Yes b) No |
| B6. How much time do you spend on social media? | a) Less than 2 hours  b) 2 to 5 hours  c)More than 5 hours. |
| B7. Do you often eat soft drinks – for example, Coca-Cola, Pepsi, Seven Up, Marinda, Mojo or something like that? | a) Yes  b) No |
| B8. Did your family/ relatives have had breast cancer? | a) Yes  b) No |
| B9. Do you use bra (bras)? | a) No (not at all)  b) Some time of day (only when going out)  c) Day and night (24 hours) |
| B10. Do you use a larger bra than is needed? | a) Yes b) No |

**SectionC: Menstruation-related Information**

| C1. At what age does the first menstruation begin? | 1. 8-11 years 2. 12-14 3. 15 years or later |
| --- | --- |
| C3. your menstruation --- | 1. Regular (regular) 2. Irregular (Irregular) |
| C4. How long does the menstruation last? | 1. Less than 3 days 2. 3-6 days 3. 7 days or more. |
| C5. The amount of blood you have during your menstruation? | 1. Comparatively less 2. Normal 3. More/ too much |
| C6. Considering the last 3/4 menstruation - what other problems would you face 2-3 weeks before the start of the menstruation? (More than one option can be taken) | 1. Waist pain 2. Bad temper 3. Unpleasantness 4. Breast pain 5. Weight increase 6. Nothing |
| C7. Considering the last 3/4 menstruation - do you have abdominal pain during the menstruation? | A) Severe pain  b) Moderate pain  c) No pain |
| C8. Did you know that someone in your family or relatives has breast pain before or after menstruation? | a) Yes  b) No |
| C9. Considering the last 3/4 of menstruations - *Do you have swelling and tenderness during or immediately preceding menstruation, as well as breast tissue pain/swelling and tenderness outside of the menstrual period*? Those whose breast does not hurt - you can end your survey here. | a) Yes  b) No |
| C10. What is the pain - | a) It happened only once.  b) It's happening again and again |
| C11. Do you have pain in both breasts? | a) Yes in two breasts.  b) Not in one breast |
| C12. And what is this pain- | a) The whole breast is in jure  b) In certain parts. |
| C13. Does fever come with breast pain? | a) Yes  b) No |
| C14. Does the breast pain go away after the start of the menstruation? | a) Yes  b) No |
| C15. Does this pain affect your daily life? | a) Yes  b) No |
| C16. Does this pain make it difficult for you to sleep? | a) Yes b) No |
| C17. How much out of 10 will you give the severity of your pain? (1 = least, 10 = most) | .......................................... |
| C18. What kind of pain is your pain? | 1. Tingling 2. Throbbing 3. Stinging 4. Burning 5. Cramping 6. Crushing 7. Tugging |
| C19. Have you gone to the doctor? | a) Yes b) No |
| C20. Check up regularly? | a) Yes b) No |
| C21. What kind of check-ups/tests have you done? (More than one option can be taken) | 1. Ultra Sono 2. Clinical test (just size shape) 3. Mammography 4. Other 5. It wasn't tested. |
